# Late-onset vascular and tumor complications after childhood brain radiotherapy: A rare case of dual sequelae

**DOI:** 10.1016/j.radcr.2026.01.063

**Published:** 2026-02-13

**Authors:** Quoc-Huy Ly, Shimon Garrel, Karthik Kanamalla, Armin Mahabadi, Daniel Masri

**Affiliations:** a College of Medicine, SUNY Downstate Health Sciences University, 450 Clarkson Ave, Brooklyn, NY 11203; b Department of Radiology, SUNY Downstate Health Sciences University, 450 Clarkson Ave, Brooklyn, NY 11203; c Department of Radiology, Maimonides Medical Center, 4802 10th Ave, Brooklyn, NY 11219

**Keywords:** Radiotherapy-induced vasculopathy, Radiotherapy-induced meningioma, Childhood brain tumor, Moyamoya

## Abstract

Radiotherapy (RT) remains a mainstay of pediatric craniopharyngioma management. The long-term sequelae of intracranial RT are well documented and include radiation-induced brain tumors, cerebral vasculopathy, cognitive impairment, and an elevated risk of cerebrovascular accidents (CVAs). Patients treated for suprasellar or intrasellar tumors are at an increased risk of vasculopathy, likely because the Circle of Willis lies within the RT field. Here, we present a rare case of a patient who, 30 years after initial RT, developed multiple progressive late effects of cranial radiation, resulting in significant morbidity. To our knowledge, few case reports have described the concurrence of both a radiation-induced meningioma and radiation-induced vasculopathy, highlighting the rarity of this presentation and underscoring the importance of lifelong surveillance in pediatric brain tumor survivors especially within the RT treatment field.

## Introduction

Radiotherapy (RT) is a commonly used treatment modality in the management of pediatric craniopharyngioma, and has been a therapeutic cornerstone for patients with recurrent disease [1]. RT treatment has been associated with increased progression free survival (PFS) in pediatric patients when compared to surgery alone, but patients also experience significant long-term morbidity [[2], [3]]. Long term intracranial sequelae of RT are common and have been reported as far back as the early 20th century [4]. Radiation-induced meningioma (RIM) is a well-documented complication of RT, with an estimated latency period from initial RT treatment to diagnosis of approximately 30 years [[4], [5]]. Childhood cancer survivors exposed to intracranial radiotherapy face a 5%-12% risk of meningioma by age 40, with risk increasing in a dose-dependent manner [[6], [7]]. Radiotherapy-induced occlusive vasculopathy (RIOV) is another well recognized sequela of RT, although the term remains poorly defined and described in the current literature [8]. RIOV has been used to describe cases of occlusive or stenotic cerebral vessel disease in the setting of RT treatment. Patients who receive cranial irradiation for supra- or intrasellar tumors are at an increased risk of RIOV, with reported incidences of 9%-20% for any vasculopathy and 5%-17.8% for severe forms including moyamoya disease when compared to patients being treated for nonsellar tumors [8]. Here, we present a rare case of a 61-year-old female who, over 30 years after receiving radiotherapy for a craniopharyngioma, was noted to have 2 distinct and progressive sequelae on neuroimaging: occlusive vasculopathy and a meningioma. While at least 1 prior report has described both radiation-induced vasculopathy and secondary intracranial neoplasm following pediatric cranial radiotherapy, the concurrent development of both conditions remains rare [9]. This case underscores the importance of long-term surveillance for childhood cancer survivors, as radiation-induced effects can cause significant morbidity decades after initial treatment.

## Case presentation

A 63-year-old female with a history of craniopharyngioma diagnosed at age 14 and treated with radiotherapy in Guyana presented to the emergency department in 2011 at age 49 with new onset bitemporal hemianopsia, bilateral cranial nerve III palsy, and new-onset right lower extremity weakness. The patient otherwise had an unremarkable medical and surgical history, which was only notable for a remote periauricular tumor resection.

**Fig. 1:**
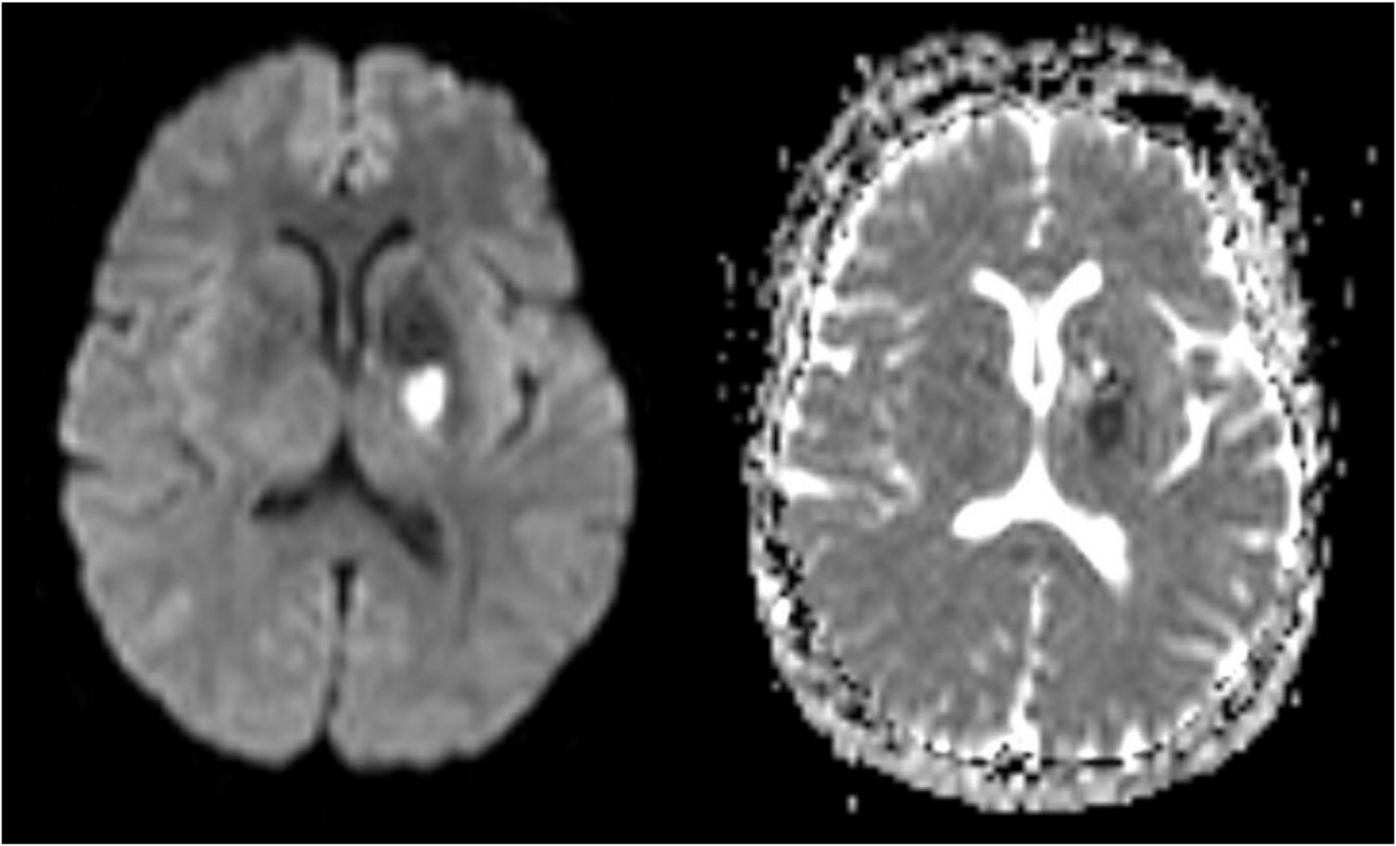
Diffusion-weighted imaging of the brain (left) and corresponding apparent diffusion coefficient (ADC) map (right) demonstrates an acute left gangliocapsular infarct diagnosed in 2011. The patient’s presenting complaint of acute-onset right lower extremity weakness was concordant with MRI findings.

**Fig. 2:**
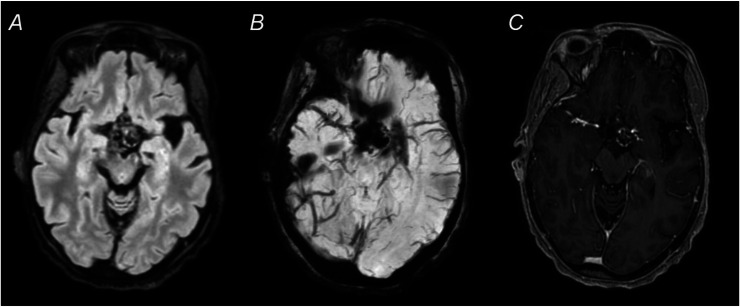
Subsequent MRI of the brain was performed with and without the administration of intravenous contrast. Axial T2-FLAIR sequence demonstrates a sellar and suprasellar mass with internal foci of low signal (A), corresponding with an area of susceptibility artifact on susceptibility-weighted imaging (SWI) sequence (B). This was compatible with the patient’s history of a treated craniopharyngioma. Mass effect on adjacent structures was causing chronic bitemporal hemianopsia and bilateral cranial nerve III palsy. Patchy enhancement was noted in the suprasellar region on axial postcontrast T1-weighted sequence (C), however follow-up over 2 decades did not demonstrate any changes suspicious for recurrent or residual tumor.

**Fig. 3:**
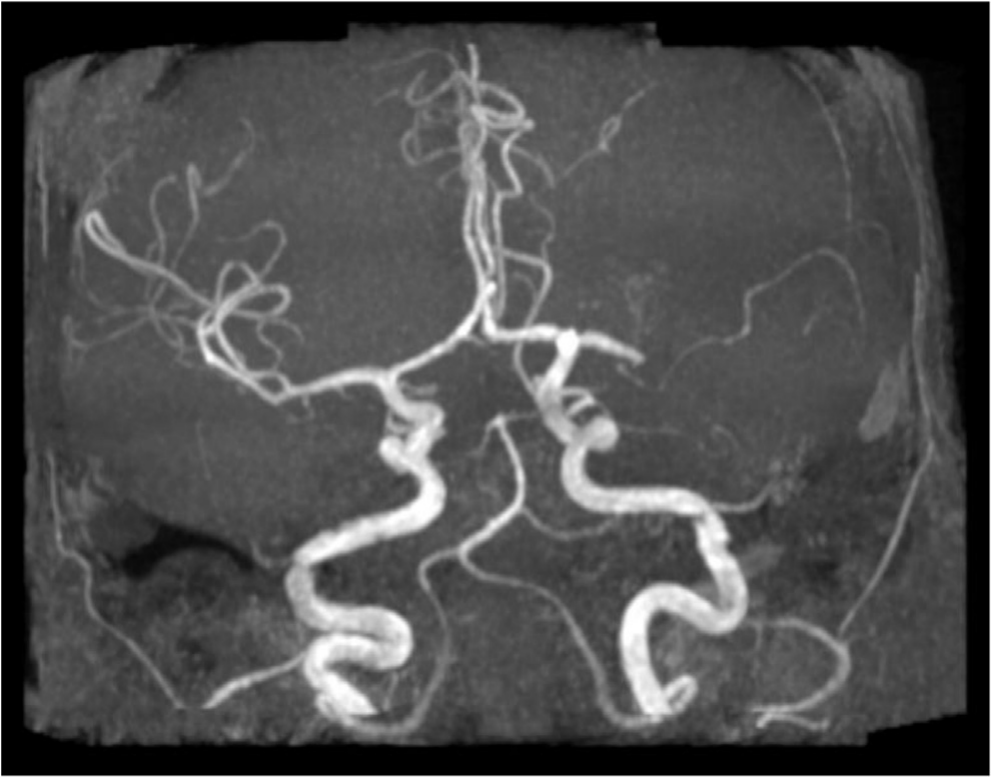
Coronal maximum intensity projection (MIP) images derived from time-of-flight (TOF) noncontrast MR angiography of the head demonstrates an occluded proximal left middle cerebral artery.

Initial noncontrast computed tomography (CT) of the head revealed findings suggestive of a subacute left gangliocapsular infarct and a calcified sellar/suprasellar mass. Additional investigation was performed with noncontrast magnetic resonance imaging (MRI), which revealed an acute left gangliocapsular infarct and a calcified sellar/suprasellar mass compressing atrophic optic pathway structures (Fig. 1, Fig. 2). The latter finding was consistent with treated craniopharyngioma. Magnetic resonance angiography (MRA) of the head and neck revealed an occluded left middle cerebral artery at the M1 segment (Fig. 3). The patient was managed medically at the time and since then has returned for interval routine neurologic follow-up.

**Fig. 4:**
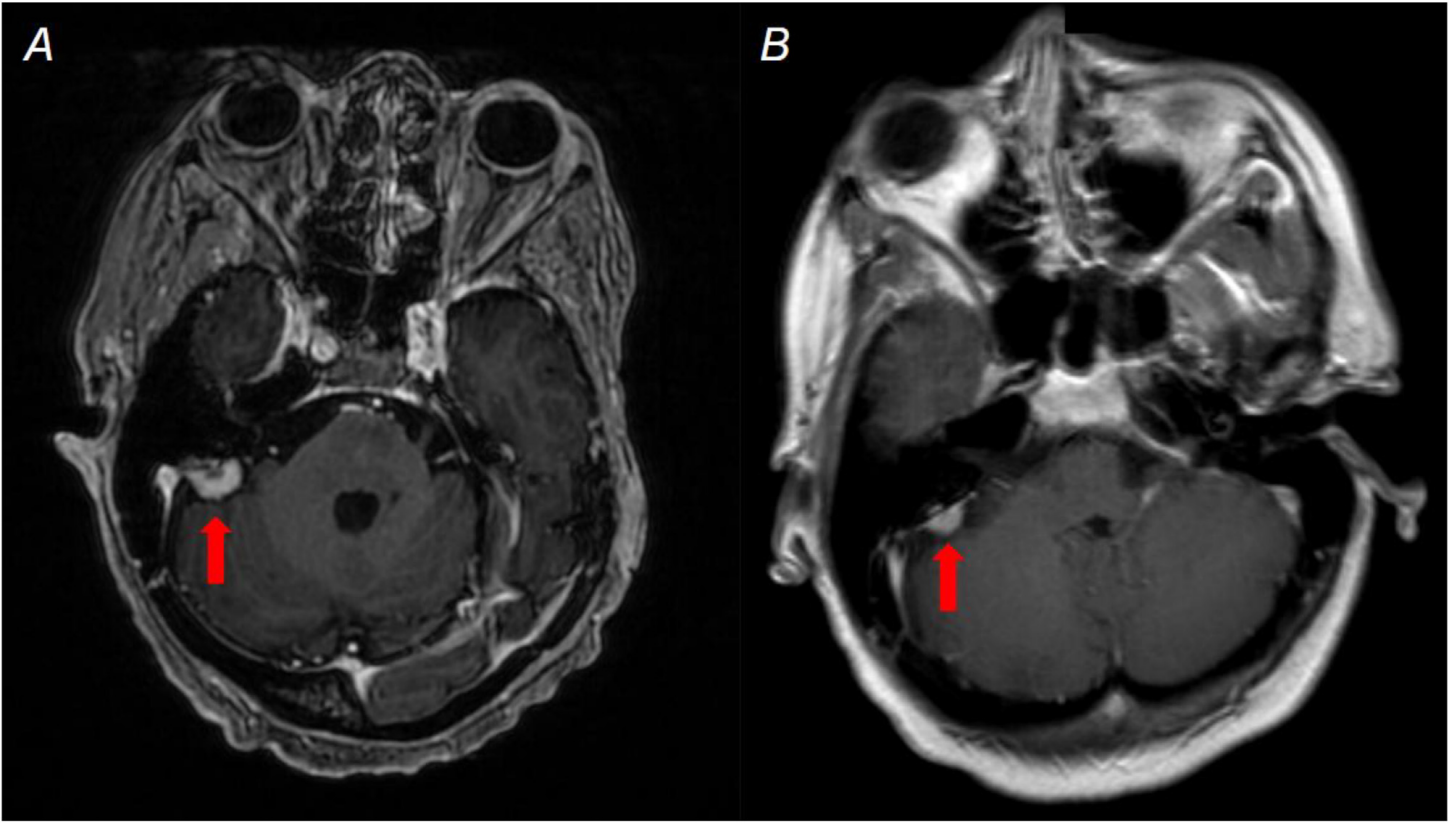
Brain MRI performed with and without contrast in 2025 shows a right petrous mass attached to the dura (red arrows). The mass demonstrates enhancement on axial postcontrast T1-weighted sequence and a dural tail, consistent with a meningioma (A). This was initially diagnosed in 2013 (B) and has been slowly growing. This was felt to reflect a radiation-induced meningioma given her history of childhood cranial radiotherapy.

Since 2011, there has been no recurrence of her craniopharyngioma; however, in 2013 she was diagnosed with a right petrous meningioma on routine follow-up brain MRI (Fig. 4). Over the past 2 decades, this meningioma has been slowly growing but exerts no mass effect on the adjacent cerebellum.

Given her history of radiation therapy as a young adult, these findings were most consistent with postradiotherapy occlusive vasculopathy and radiation-induced meningioma. Clinically, the patient has been noted to have progressively worsening neurologic symptoms over the years, including cranial nerve III palsy, visual disturbances, dysarthria, and vertigo. These symptoms are likely secondary to both the slow growth of the meningioma and the patient’s underlying vasculopathy. According to available documentation, the patient was offered radiosurgery as a potential treatment option for the meningioma in May 2025; however, they have elected to continue with routine monitoring as of the current time of this report (Table 1).

**Table 1:** Event timeline.

Patient age	Year	Event/Diagnosis	Significance
14	∼1976	Craniopharyngioma diagnosis	Treated with radiotherapy
49	2011	Acute neurologic presentation	New onset bitemporal hemianopsia, CN III palsy, right lower extremity weakness
	2011	Imaging findings (CT/MRI/MRA)	Acute left gangliocapsular infarct secondary to left MCA M1 occlusion. Sellar mass consistent with treated tumor.
51	2013	New diagnosis on routine MRI	Right petrous meningioma detected (radiation-induced lesion)
51–63	2013 - 2025	Follow-up course	Slow growth of meningioma. No craniopharyngioma recurrence. Progressive worsening of existing neurologic symptoms.
63	2025	Current status	Continued neurologic follow-up.

## Discussion

In patients with a history of cranial radiotherapy who later present with occlusive vasculopathy, the differential diagnosis includes radiation-induced vasculopathy, atherosclerotic disease, and focal arterial dissection. Given this patient’s remote history of RT, the chronic and progressive pattern of vascular stenosis, and the lack of traditional atherosclerotic risk factors or imaging evidence of arterial dissection or other mechanical causes of luminal compromise, radiation induced disease remains the most plausible etiology.

Although we were unable to obtain the patient’s original medical records from her prior treatment in Guyana, it is highly likely that her radiation course used early 2D orthogonal beam techniques, with critical structures shielded using lead or cerrobend blocks [10]. These rudimentary methods required substantially larger treatment fields because accurate beam placement and conformality were limited in a 2D planning environment [10]. Given the likelihood of wide field exposure during craniopharyngioma treatment in the 1970s, the anatomic distribution of her current pathology, encompassing both skull base meningeal tissues and the proximal MCA circulation, aligns closely with expected regions of collateral radiation dose.

Taken together, the long latency period, the pattern of injury, and the known limitations of historical radiation techniques provide compelling circumstantial evidence that both the meningioma and the large vessel stenosis represent delayed radiation induced sequelae rather than unrelated de novo processes.

This case is also notable as disparities in radiotherapy access and quality are pronounced in resource-limited countries, where insufficient equipment, inadequate staffing, limited supportive infrastructure, and a relative lack of multidisciplinary decision-making such as tumor boards negatively affect patient outcomes [[11], [12]]. Moreover, long-term surveillance for late radiation effects is often less feasible in resource-limited settings, which may have contributed to the late recognition and management of sequelae such as those of this patient [13]. Beyond these systemic challenges, the discontinuity of care that arises when treatment is started in 1 country and follow-up occurs in another over several decades further complicates providers’ ability to continuously monitor and manage care.

Moyamoya vasculopathy is a rare condition characterized by progressive stenosis or occlusion of the distal internal carotid arteries and their branches. The condition is named after the Japanese term “moyamoya,” which describes the “puff-of-smoke” appearance created by the formation of collateral vessels secondary to large vessel occlusion. Presenting symptoms may vary but include transient ischemic attacks and strokes, hemorrhagic neurological events, as well as headaches (migraines being the most common headache type) and neurocognitive impairment [14].

The American Heart Association distinguishes moyamoya disease, which is idiopathic, from moyamoya syndrome, in which patients with moyamoya disease also have a related comorbid condition. In this case, our patient had both a history of a brain tumor as well as head irradiation, but other associated disorders with moyamoya syndrome include autoimmune disease, Down syndrome, and neurofibromatosis type 1 [14]. However, other authors have used the term “occlusive radiation vasculopathy” to describe similar cases, as the characteristic collateral vessels in moyamoya may not always be seen on angiography, as in this patient case [15]. Diamox (acetazolamide) challenge testing can be used as a follow-up diagnostic study in suspected moyamoya, as it evaluates cerebrovascular reserve by assessing the ability of cerebral vessels to increase blood flow after induction by a vasodilator; a lack of increased perfusion suggests hemodynamic compromise, and can inform medical versus surgical management options [15].

Radiation-induced moyamoya vasculopathy has been reported in multiple series, with higher incidence in patients who were younger at the time of radiotherapy, as well as with increasing dose of radiation [16]. Meta-analysis demonstrates a dose-response relationship, with rates of cerebrovascular toxicity of the Circle of Willis of 0.2% at 30 Gy, 1.3% at 45 Gy, and 4.4% at 54 Gy, with shorter latency periods associated with higher radiation exposure levels [17].

Radiation-induced meningiomas (RIMs) represent the most common secondary neoplasm after cranial irradiation. They are more likely to occur as more than 1 lesion, present atypically or aggressively, and carry higher recurrence rates after resection compared to sporadic meningiomas [[18], [19]]. Studies suggest that the prevalence of RIMs is increasing, likely due to improved long-term survival rates among pediatric cancer patients, many of whom are treated with intracranial radiotherapy [20]. While surgical resection remains a mainstay of treatment for symptomatic or rapidly enlarging lesions, conservative management and monitoring can be done for more indolent tumors, as is in this case. That said, even stable-appearing RIMs should be observed in the long term due to the risk of acute growth acceleration and increased tumor burden [[18], [19]].

The prognosis in cases of radiation-induced moyamoya and meningioma varies depending on multiple factors, including cerebrovascular reserve, tumor growth rate, and neurological symptoms/progression. This patient’s clinical course, marked by progressive cranial neuropathies, visual decline, dysarthria, and vertigo, reflects not only the significant long-term impacts of radiotherapy performed decades previously to manage a pediatric brain tumor, but also the importance of persistent follow-up of such patients to detect and manage both vascular and neoplastic sequelae.

## Conclusion

This report describes a case where both occlusive radiation vasculopathy and radiation-induced meningioma were present in a patient over 40 years since the diagnosis and treatment of a pediatric craniopharyngioma with intracranial radiotherapy. These findings, identified after the onset of multiple neurologic symptoms, support the significance of long-term surveillance for vascular and neoplastic complications in childhood cancer survivors. This case also provides additional considerations regarding the cost-benefit of radiotherapy interventions for the short-term management of childhood brain cancer versus remote sequelae of such treatment.

## Declaration of generative AI and AI-assisted technologies in the manuscript preparation process

During the preparation of this work the authors used OpenAI’s ChatGPT in order to improve the report’s language and readability. After using this tool/service, the authors reviewed and edited the content as needed and take full responsibility for the content of the published article.

## Patient consent

All identifying data and details that could be linked to the patient were removed from the details of this report and the images used to present the case.
